# Periodontal Disease and Late-Onset Aortic Prosthetic Vascular Graft Infection

**DOI:** 10.1155/2015/768935

**Published:** 2015-01-21

**Authors:** Stephanie Thomas, Jonathan Ghosh, Johnathan Porter, Adele Cockcroft, Riina Rautemaa-Richardson

**Affiliations:** ^1^Department of Microbiology, University Hospital of South Manchester, Wythenshawe Hospital, Southmoor Road, Manchester M23 9LT, UK; ^2^Department of Vascular Surgery, University Hospital of South Manchester, Wythenshawe Hospital, Southmoor Road, Manchester M23 9LT, UK; ^3^The University of Manchester, Manchester Academic Health Science Centre, Institute of Inflammation and Repair, Oxford Road, Manchester M13 9PT, UK

## Abstract

Prosthetic vascular graft infection (PVGI) is a rare but significant complication of arterial reconstructive surgery. Although the relative risk is low, the clinical consequences can be catastrophic. Microbiological data on causative bacteria are limited. We present four cases of late-onset PVGI. Using a culture-independent nucleic acid amplification method for analysis of intraoperative samples, the presence of bacteria highly suggestive of an oral source was reported. Examination by an oral health specialist confirmed the presence of chronic periodontal disease. We hypothesize that chronic oral infection may be a previously unreported risk factor for the development of late-onset PVGI.

## 1. Introduction

Prosthetic vascular graft infection (PVGI) has an incidence of 1–6%, an associated morbidity of 40–70%, limb amputation of up to 70%, and a recognised mortality of 30–50% [1].

Microbiological data on organisms associated with PVGI are rarely published, mainly due to the need for early initiation of broad-spectrum antimicrobial therapy before graft excision and reconstruction. The aim is to disinfect the vascular bed, but this decreases the sensitivity of routine laboratory culture based methods. In addition, PVGIs are associated with polymicrobial biofilm formation around the graft [2]. Bacteria growing within the biofilm disperse sporadically, whereby preoperative blood cultures are unlikely to be positive. Blood cultures are more likely to be positive in patients with an aortodigestive fistula, which provides gut microorganisms with a constant portal of entry into the blood stream.

Epidemiological data on PVGI are similarly poorly defined, but reported risk factors for late-onset infections (>4 months after primary surgery [1]) include postoperative or early wound infection, wound complications at any time, and the presence of a femoral incision [3].

We report four cases where odontogenic bacteria have been implicated in late-onset aortic PVGI, using culture-independent nucleic acid amplification. To our knowledge, this has not been previously reported.

## 2. Case Reports

We present four cases of late-onset aortic PVGI, diagnosed and treated at our tertiary referral Vascular Surgical Unit between February and May 2013.

Case 1 was a 46-year-old female who presented three years after redo aortobifemoral graft (initial graft insertion, twelve years previously), with bleeding from an opening in the right groin. A CT angiogram aorta carried out on admission reported an infected and occluded aortic bifurcation graft (gas seen along the right limb of the graft) and a probable thrombosed aortoenteric fistula.

Case 2 was a 69-year-old male who presented one year after endovascular aneurysm repair (EVAR), with sudden severe back and abdominal pain and melaena. A CT angiogram aorta reported that a previously noted endoleak had increased significantly with gas seen within the anterior native aortic sac. In addition, the duodenum was reported to be closely applied to the anterior portion of the aneurysm with marked inflammatory stranding around the native aortic aneurysm sac. Appearances were consistent with an aortoenteric fistula.

Case 3 was a 57-year old female who was admitted to our unit three years post axillo-bi-femoral reconstruction following explantation of an infected aortobifemoral graft. She presented with pus discharging from the left groin. A CT angiogram aorta reported a focal collection around the left femoral branch of the graft with an area of infiltration of the adjacent fat likely to represent a focal graft infection.

Case 4 was an 80-year-old male who presented seven years after aortic aneurysm repair with back pain and fever. In view of the prosthetic material in situ, he was admitted to the medical assessment unit and a CT scan was carried out which showed small bowel adherence to the distal aortic graft with gas circling the graft suggestive of an aortoenteric graft fistula.

Cases 1–3 underwent explantation of the infected graft, with debridement of infected tissue and vascular reconstruction. Where debridement was carried out, intraoperative samples were collected and sent for microbiological analysis. In view of multiple comorbidities, case four was unfit for surgical intervention; however, multiple sets of blood cultures were collected on admission.

## 3. Diagnosis

In addition to routine laboratory culture, the intraoperative samples collected from cases 1–3 were sent for 16S rDNA real-time PCR identification to the Department for Bioanalysis and Horizon Technologies at the Centre for Infections, Colindale, London. In each case, the 16S rDNA analysis detected the presence of a mixture of bacteria highly suggestive of an oral source.

In case 1, analysis of aortic graft fluid, the sequence data displayed a series of overlapping peaks indicative of a mixture of bacteria. Mixed sequence trace file analysis showed that the mixture in the sample was too complex to accurately determine which bacterial species were present. However, it was suggestive of a mixture of oral anaerobic bacteria.

In case 2, aortic thrombus tissue, a 16S rDNA PCR product, was obtained, indicating the presence of a mixture of bacteria in the sample. However, it was not possible to delineate the species. Mixed trace file analysis showed presence of oral anaerobic bacteria including* Fusobacterium *sp.,* Prevotella *sp., and* Propionibacterium avidum.*

In case 3, groin pus detected* Pediococcus pentosaceus*. Admission blood cultures collected in case 4 also isolated organisms of oral aetiology, including* Streptococcus oralis*,* Lactobacillus* species,* Klebsiella pneumonia,* and* Enterococcus faecium*.

In view of the microbiology detected, a postoperative clinical oral examination by a specialist in oral medicine was arranged for cases 2, 3, and 4 who remained in-patients in the unit. Case 2 had evidence of extensive past need for dental care, a number of missing teeth and various dental restorations, and clear clinical signs of past chronic periodontitis. Case 3 was in acute need for dental care with snapped teeth, large carious lesions, and chronic periodontal infection. A number of teeth were missing; partial dentures were in use and moderate denture stomatitis was detected in the underlying mucosa. Case 4 had evidence of extensive past need for dental care including root treatments and crowns and clinical signs of chronic periodontitis. Radiological imaging of the jaws by orthopantomography was carried out in two patients (cases 2 and 3). This was reviewed by a maxillofacial radiologist, confirming the presence of severe periodontal disease in both cases.

## 4. Discussion

Broad-range 16S ribosomal DNA gene polymerase chain reaction (PCR) is a molecular based technique used for detection and identification of bacterial pathogens in clinical specimens from patients with a high suspicion for infection. Unlike routine culture methods, molecular assays detect bacterial genetic material (DNA). They do not rely on growth of an organism and so results are unaffected by prior antimicrobial use.

It is well known that the oral cavity represents an important reservoir of microorganisms [4]. There have been well-documented associations between periodontal disease and, for example, infective endocarditis, brain, liver and lung abscess, and Lemierre's disease. Low-grade infections can persist asymptomatically and remain dormant for years. In patients with good oral health, only small numbers of mostly facultative bacterial species enter the bloodstream. This occurs following any form of dental manipulation, even daily activities such as eating and tooth brushing [5]. With poor oral hygiene, however, the number of bacteria colonizing the teeth increases, potentially introducing more bacteria into tissues and the bloodstream, leading to an increase in the prevalence and magnitude of bacteraemia [6]. Small numbers of bacteria become quickly cleared by the reticuloendothelial system, but the more intense the bacteraemia, the greater the risk for dissemination and metastatic infections. This is of particular concern for patients with prosthetic material in situ. In line with this, in a recent study, DNA of bacteria typically causing root canal infection was detected in 78.2% of thrombus aspirates of patients with myocardial infarction [7].

In recent years, the role of oral bacteria in endovascular infections has been widely debated. Present United Kingdom National Institute for Health and Care Excellence (NICE) guidance now recommends that the benefits from antibiotic prophylaxis for patients undergoing dental procedures, who are at risk of infective endocarditis, for example, are outweighed by the risks of possible adverse effects to the patient and of antibiotic resistance developing. This guidance is based on one clinical trial of antibiotic prophylaxis in the prevention of infective endocarditis. Patients with prosthetic vascular grafts in situ, who are at subsequent risk of PVGI following dental manipulation, were not included in the cohort [8]. Interestingly, a recent study looking at incidence of infective endocarditis in England from 2000 to 13 shows that prescriptions of antibiotic prophylaxis have fallen substantially and the incidence of infective endocarditis has increased significantly since the introduction of the 2008 NICE guidelines [9].

Successful management of an infected vascular prosthesis remains a significant, occasionally insurmountable challenge. If excision of the infected graft is not possible, patients need broad-spectrum antibiotics for prolonged periods of time, often chosen empirically due to the lack of microbiological data available and often lifelong, with the associated risks of adverse events that include* Clostridium difficile* infection, development of resistance, and treatment failure secondary to poor compliance. For patients undergoing surgery for an infected aortic prosthesis, high operative mortality (reportedly in the range of 10–20% for graft excision [10]) and morbidity, together with the lack of clear national consensus treatment guidelines, are constant reminders that prevention of infection should be the single priority in vascular graft surgery.

Our patients represent a small series and oral health was examined retrospectively. However, the presence of bacteria of an oral source obtained from intraoperative tissue samples and blood, collected from 4 patients with infected aortic grafts and confirmed periodontal disease, suggests revisiting the role of odontogenic bacteria in endovascular infection. Unfortunately, patient 1 died quite soon after the primary sampling and not all investigations were done in his case. Quantitative analysis of bacterial DNA was unavailable and the oral health status was not obtained. In this case, a sample of oral bacteria from either saliva or supra- or subgingival plaque in order to confirm the oral origin of these microorganisms may have been helpful. This may also be a helpful addition to any further studies going forward. In view of the catastrophic consequences of infection, it may be reasonable to recommend a simple presurgical oral assessment for all patients undergoing complex elective aortic graft surgery. In this way, any underlying periodontal disease can be addressed before surgery, the need for good oral hygiene postoperatively can be reinforced, and bacterium of oral origin can be covered when choosing antimicrobial prophylaxis.

In summary, we report four cases where odontogenic bacteria have been associated with late-onset aortic PVGI, using culture-independent nucleic acid amplification. We hypothesize that chronic oral infection may be a risk factor for the development of late-onset PVGI. To our knowledge, this has not been previously reported. We acknowledge that further evaluation of this theory is warranted.
